# Prevalence of obesity in primary care using different anthropometric measures – Results of the German Metabolic and Cardiovascular Risk Project (GEMCAS)

**DOI:** 10.1186/1471-2458-8-282

**Published:** 2008-08-11

**Authors:** Hans Hauner, Peter Bramlage, Christian Lösch, Elisabeth Steinhagen-Thiessen, Heribert Schunkert, Jürgen Wasem, Karl-Heinz Jöckel, Susanne Moebus

**Affiliations:** 1 Else Kröner-Fresenius-Zentrum für Ernährungsmedizin, Technical University Munich, Klinikum rechts der Isar, Ismaninger Str. 22, 81675 München, Germany; 2 Institute for Clinical Pharmacology, Medical Faculty, Technical University of Dresden; Fiedlerstrasse 27, 01307 Dresden, Germany; 3 Institute for Medical Informatics, Biometry and Epidemiology, University Hospital, University of Duisburg-Essen, Hufelandstrasse 55, 45122 Essen, Germany; 4 Charité, Campus Virchow Klinikum, Augustenburger Platz 1, 13353 Berlin, Germany; 5 Medical Clinic II, University Hospital Schleswig-Holstein, Campus Lübeck, Ratzeburger Allee 160, 23538 Lübeck, Germany; 6 University of Duisburg-Essen, Schützenbahn 70, Eingang Waldthausenstr., 45127 Essen, Germany

## Abstract

**Background:**

Obesity is one of the greatest challenges in primary health care. The BMI describes fat mass and waist circumference (WC) fat distribution and total metabolic and cardiovascular risk. It was aim of the present study to assess the prevalence of a) overweight and obesity and b) an increased and high WC in adults seeking primary care in Germany and to describe the associations of both measures with cardiovascular risk factors and prognosis.

**Methods:**

This was a point prevalence study with 1,511 primary care physicians and 35,869 adult patients in 2005. Bodyweight, height and waist circumference was measured and blood samples taken to determine the presence of cardiovascular risk factors, including lipids, blood pressure, fasting glucose, low physical activity, smoking and family history of myocardial infarction. We calculated rate ratios stratified for age and gender.

**Results:**

There was a high prevalence of overweight (45.7% male [95%CI 44.9–46.5]; 30.6% female [95%CI 30.0–31.2]) and obesity (24.7% male [95%CI 24.0–25.4]; 23.3% female [95%CI 22.8–23.9]). 36.4% of male [95%CI 35.6–37.2] and 41.5% of female [95%CI 40.8–42.1] had a high WC (male > 102, female > 88 cm). A high WC in addition to an overweight BMI identified patients with more risk factors (male: mean of 3.93 risk factors (RF) at a WC > 102 cm vs. 2.88 RF in patients ≤ 94 cm; female 3.58 RF at a WC > 88 cm vs. 2.41 RF ≤ 80 cm).

**Conclusion:**

There is a high prevalence of obesity (24.7% of male and 23.3% of female) and, in particular, abdominal obesity (36.4% of male and 41.5% of female) in adults attending a primary care physician in Germany. The determination of the BMI is sufficient to assess risk in normal weight and obese patients, while a high WC identifies high risk patients from within the overweight group.

## Background

Overweight and obese persons have an increased prevalence of cardiometabolic risk factors compared to patients with normal weight, especially when they are abdominally obese. Different anthropometric measures like weight, height, waist- and/or hip circumference, direct measurements of abdominal fat using MRI scans and various computations of these variables have been proposed to identify patients with an increased risk for developing diabetes and cardiovascular events [1].

*Body mass index (BMI) *is the most widely accepted measure of obesity in populations and in clinical practice. Therefore, most reports on the prevalence of obesity and its trends over time and distribution among regions are based on the determination of BMI. Using this definition the World Health Organization (WHO) estimated that over 1 billion people are overweight globally, and if the current trend continues, that this number will increase to 1.5 billion by 2015 [2]. Previous studies reported rates of 38% for overweight and 20% for obesity in the adult population in Germany [3,4].

While the BMI is, with limitations, generally a proper measure of total body fat measuring the *waist circumference *provides insight into the distribution of body fat. It discriminates persons with abdominal obesity from those with a more gluteal-femoral fat deposition. Abdominal obesity has been shown to be associated with an increased cardiovascular risk [5-8] and its use as the central variable in defining persons with the metabolic syndrome highlights its role as a risk indicator. Generally, a cut-off of > 102 cm in men and > 88 cm in women (which is predicting obesity in Scottish people [9]) is accepted indicating a clearly elevated health risk, but already cut-offs of > 94/> 80 cm (termed abdominal overweight, which is predicting overweight [9]) have been found to be associated with a moderately increased cardiovascular risk among Caucasians [10].

Primary care physicians are besides public health initiatives the most promising party to improve the management of overweight and obesity since they are the gatekeepers of the healthcare system in most countries. Therefore, nationwide data from the primary care sector are needed to recognize the dimension of this public health problem and to develop strategies to handle it. The present analysis is based on the dataset of the "German Metabolic and Cardiovascular Risk Project" (GEMCAS), which is a nationwide point prevalence study in 35,869 adult primary care attendees in Germany [11]. Aim of this study was to assess the prevalence of overweight and obesity and the prevalence of an increased waist circumference in primary care and to study the relationship of these anthropometric variables to the presence of cardiovascular risk factors in defined subpopulations.

## Methods

### Study design and participating physicians

The design of the present study has been described earlier [11]. It was a 2-week cross-sectional prevalence study at General practitioners' and internists' offices using a stratified, randomized sampling method for physician selection. Ethical approval was granted from the local ethics committee of the University Hospital of the University Duisburg-Essen, Germany.

### Study population

The study population comprised consecutive patients with an age of 18 years and above with either gender who visited their GP at the participating sites on the day of the survey. The only reasons for exclusion from the study were conditions that made it impossible for the patient to participate (serious disabilities or diseases), acute emergencies, or pregnancies and breast-feeding within the previous 3 months. Participating GPs and their staff assessed the patients with a standardized questionnaire with a focus on cardiovascular risk factors and basic data on sociodemographic as well as anamnestic information and life style. In total, 1,511 general practices (8.75% of initially contacted random sample of 17,271 physicians) from 397 out of 438 German cities and administrative districts enrolled 35,869 patients (age range: 18–99 years, women 61.1%).

### Diagnostic procedures

*Body weight and height *were provided by the treating physician (or nurse) indicating whether measured (1/3^rd ^of cases) or anamnestic data (about 2/3^rd^) were used. Overweight was defined as a Body Mass Index (BMI) of ≥ 25 and < 30 kg/m^2^; obesity as a BMI of ≥ 30 kg/m^2^. *Waist circumference *(WC) was measured with a common tape provided to all physicians and measured midway between the last rib and the highest part of the iliac crest in a standardized manner. A high WC was diagnosed > 102 cm in men and > 88 cm in women. *Blood glucose: *Initial capillary blood glucose (BG) quick test was performed independent of the fasting status to identify all patients with a BG concentration of < 5.6 mmol/L or those with a BG concentration of ≥ 11.1 mmol/L. Patients with a non-fasting BG level of 5.6 mmol/L and < 11.1 mmol/L were scheduled for a follow-up visit within the following 2 weeks for a second fasting blood sample. Additionally, venous blood samples were collected and analyzed for levels of glucose, LDL-cholesterol, HDL-cholesterol, total cholesterol and triglycerides using enzymatic assays (Roche Hitachi MODULAR Systems) in a central laboratory.

### Calculation of the PROCAM and SCORE cardiovascular risk scores

The PROCAM Score was used to calculate the 10-year risk for cardiovascular morbidity [12]. The score is computed using the following 8 independent risk variables, ranked in order of importance: age, LDL-cholesterol, smoking, HDL-cholesterol, systolic blood pressure, family history of premature myocardial infarction, diabetes mellitus, and triglycerides. Analyses were also done using the SCORE classification [13]. The following parameters were used: sex, age, total cholesterol, systolic BP, and smoking status.

### Statistical analyses

For the main variables of the study, basic descriptive statistics, number of observations, mean, standard deviation, median, complemented by 95% confidence intervals for prevalences were calculated. We calculated prevalence rate ratios (PRRs) stratified for age and gender. All statistical analyses were conducted using the statistical software package SAS 9.1 (SAS Institute, Cary, NC, USA) [14].

## Results

### Sample characteristics

35,869 patients from the GEMCAS study were the basis for the present analysis. Baseline characteristics have been reported previously [11,15]. In short, 61.1% of patients were female; mean age was 51.7 ± 16.1 years with an average BMI of 27.0 ± 5.2 kg/m^2^. 54.2% were smokers either presently (25.1%) or in the past (29.1%). Any cardiovascular disease was present in 16.3% of patients (n = 5,535) with a history of myocardial infarction or acute coronary syndromes being the most frequent diagnoses (n = 1,968, 12.3%).

### Prevalence of overweight using BMI and WC thresholds

**Figure 1:**
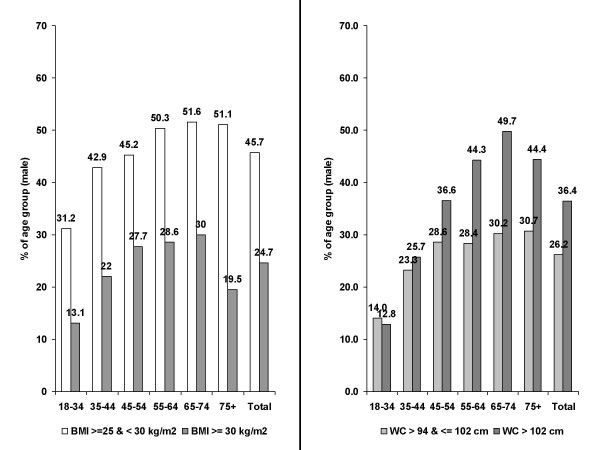
Body Mass Index (BMI) and Waist Circumference (WC) in male primary care patients by age groups (in %).

In men, the prevalence of overweight was 45.7% and of obesity 24.7% (Figure 1 and Table 1). There was an age-dependent increase in BMI from the age group 18–34 until 65–74 with a slight decline in the prevalence of overweight (-0.5%) and a substantial decline of obesity (-11.1%) thereafter. 26.2% of patients had moderately increased waist circumference (WC > 94 & ≤ 102 cm), 36.4% had a high WC (> 102 cm). Again, there was an age-related increase until the age group 65–74; the proportion of patients with an increased WC was comparable in the elderly, while the prevalence of a high WC dropped by 5.3%.

**Table 1:** Overweight and obesity (BMI) and waist circumference (WC) in primary care

	BMI				WC			
	25,0 – 29,9 kg/m^2^		≥ 30,0 kg/m^2^		> 94 & ≤ 102 cm (♂) > 80 & ≤ 88 cm (♀)		> 102 cm (♂) > 88 cm (♀)	

	%	95%CI	%	95%CI	%	95%CI	%	95%CI

18–34								
male	31.2	[29.1;33.3]	13.1	[11.6;14.7]	14.0	[12.5;15.7]	12.8	[11.4;14.4]
female	18.0	[16.8;19.3]	12.8	[11.7;13.9]	14.2	[13.1;15.4]	19.2	[18.0;20.6]
35–44								
male	42.9	[40.9;45.0]	22.0	[20.2;23.7]	23.3	[21.5;25.1]	25.7	[24.0;27.6]
female	24.5	[23.2;25.8]	16.7	[15.6;17.8]	20.1	[18.9;21.4]	27.9	[26.6;29.3]
45–54								
male	45.2	[43.4;47.0]	27.7	[26.1;29.3]	28.6	[27.0;30.3]	36.6	[34.8;38.3]
female	30.6	[29.3;31.9]	24.5	[23.3;25.7]	21.8	[20.7;23.0]	40.5	[39.1;41.9]
55–64								
male	50.3	[48.5;52.1]	28.6	[27.0;30.3]	28.4	[26.8;30.0]	44.3	[42.5;46.1]
female	36.0	[34.5;37.5]	30.3	[28.9;31.8]	23.0	[21.7;24.3]	52.6	[51.0;54.2]
65–74								
male	51.6	[49.7;53.5]	30.0	[28.2;31.8]	30.2	[28.5;32.0]	49.7	[47.8;51.6]
female	40.3	[38.6;42.0]	33.2	[31.6;34.9]	21.7	[20.2;23.1]	63.2	[61.5;64.9]
75+								
male	51.1	[48.2;54.0]	19.5	[17.3;21.9]	30.7	[28.0;33.4]	44.4	[41.5;47.3]
female	41.2	[39.0;43.5]	24.5	[22.5;26.5]	24.1	[22.1;26.1]	58.3	[56.0;60.6]
Total								
male	45.7	[44.9;46.5]	24.7	[24.0;25.4]	26.2	[25.5;26.9]	36.4	[35.6;37.2]
female	30.6	[30.0;31.2]	23.3	[22.8;23.9]	20.6	[20.1;21.1]	41.5	[40.8;42.1]

**Figure 2:**
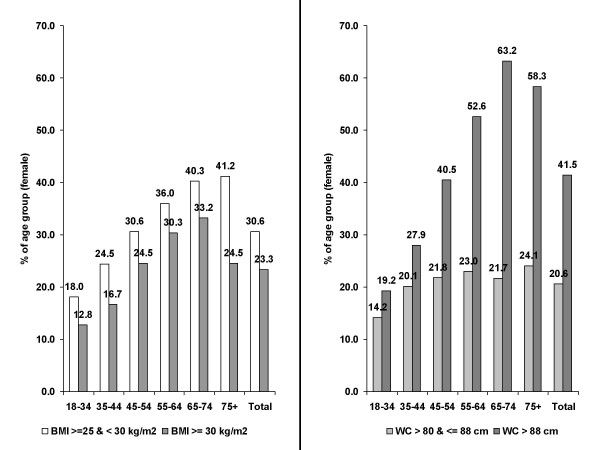
Body Mass Index (BMI) and Waist Circumference (WC) in female primary care patients by age groups (in %).

Figure 2 and Table 1 show the respective prevalence rates of anthropometric variables in women. The prevalence of obesity was 23.3% and comparable to male while the rate of overweight was substantially lower than in male (30.6% in women vs. 45.7%). Again, there was an age-related increase in prevalence with a decline in the prevalence of obesity beyond an age of 74 years. A high WC (> 88 cm) was substantially more frequent (41.5%, total) than obesity with a peak in the age group 65–74 years (63.2%). The prevalence rates of an increased WC (> 80 and ≤ 88 cm) were comparable between age groups (between 20.1 and 24.1%) except in the young (14.2%).

### Proportion of patients with an increased WC stratified by BMI subgroups

**Figure 3:**
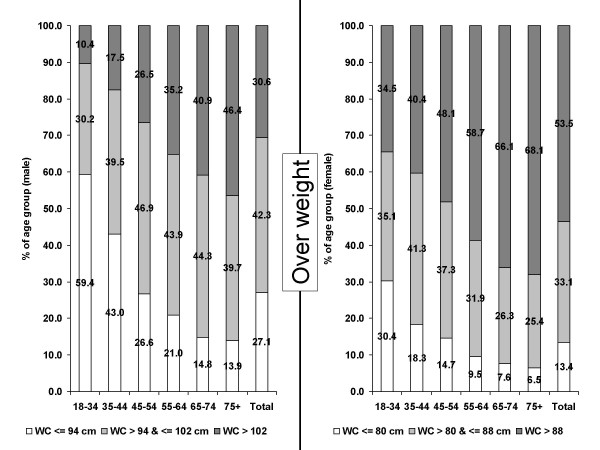
Proportion of patients with a normal, increased and high WC in the overweight BMI group (in %).

Most obese patients had a high waist circumference (87.9% of male and 94.7% of female patients) with lower rates in the young (71.1% of male, 87.8% of female). Most normal-weight patients had a normal waist circumference (83.5% of male and 73.0% of female patients) with a decline in the elderly (60.2% male, 42.5% female). However, only 39.8% of normal-weight men and 57.4% of normal-weight women in the age group beyond 74 had a normal or only elevated WC. Patients in the overweight BMI group Figure 3 had the greatest heterogeneity in WC. 27.1% of overweight men had a normal WC, 42.3% an increased and 30.6% a high WC (women 13.4, 33.1, 53.5%). The proportion of overweight patients with a normal WC was lowest in the highest age group (13.9% male, 6.5% female). On the other hand, young overweight male patients had a normal waist circumference in 59.4% (female 30.4%).

### Cardiovascular risk factors in overweight patients depending on WC

Given the diversity of overweight patients in terms of WC it seemed to be of particular importance to examine whether these patients also differ in the prevalence of cardiovascular risk factors. The risk factors assessed are displayed in table 1. In both genders, a high WC was associated with an increased prevalence of high triglycerides (> 150 mg/dL), a high blood pressure (> 140/90 mmHg), an increased fasting plasma glucose (> 100 mg/dL), the presence of diabetes mellitus and the mean number of risk factors (for individual figures see Table 2 &3). A family history of myocardial infarction on the other hand was not more frequent in patients with a high WC as compared to patients with a normal WC. The prevalence of smoking was significantly lower in overweight men with an elevated and high WC but not in women.

**Table 2:** Cardiovascular risk factors in overweight men split into WC categories

	Men BMI ≥ 25 & < 30 kg/m^2^				
	≤ 94 cm	> 94 & ≤ 102 cm		> 102 cm	

	%	%	PRR [95%CI]	%	PRR [95%CI]

LDL > 115 mg/dl					
< 45 (yrs)	60.93	67.75	1.11 [1.03;1.21]	68.44	1.12 [1.01;1.25]
45–64 (yrs)	70.36	72.91	1.04 [0.98;1.10]	68.79	0.98 [0.91;1.05]
≥ 65(yrs)	64.31	63.07	0.98 [0.89;1.09]	59.95	0.93 [0.84;1.03]
Total	65.15	68.74	1.06 [1.01;1.10]	64.95	1.00 [0.95:1.05]
TG > 150 mg/dl					
< 45 (yrs)	38.68	53.24	1.38 [1.22;1.55]	60.62	1.57 [1.37;1.80]
45–64 (yrs)	46.35	50.95	1.10 [1.00;1.21]	59.79	1.29 [1.17;1.42]
≥ 65(yrs)	34.98	43.76	1.25 [1.05;1.49]	49.69	1.42 [1.19;1.69]
Total	41.04	49.17	1.20 [1.12;1.28]	55.56	1.35 [1.26;1.45]
RR ≥ 140/90 mmHg					
< 45 (yrs)	15.31	19.03	1.24 [0.98;1.58]	26.32	1.72 [1.31;2.26]
45–64 (yrs)	24.03	29.00	1.21 [1.03;1.41]	34.71	1.44 [1.23;1.70]
≥ 65(yrs)	36.40	37.99	1.04 [0.87;1.25]	40.00	1.10 [0.92;1.31]
Total	22.20	29.74	1.34 [1.20;1.49]	36.00	1.62 [1.46;1.80]
Low phys. activity					
< 45 (yrs)	64.41	74.64	1.16 [1.08;1.25]	87.05	1.35 [1.26;1.45]
45–64 (yrs)	68.54	74.82	1.09 [1.03;1.16]	80.77	1.18 [1.11;1.25]
≥ 65(yrs)	68.63	74.78	1.09 [1.00;1.19]	81.42	1.19 [1.09;1.29]
Total	66.71	74.77	1.12 [1.08;1.17]	81.79	1.23 [1.18;1.28]
HDL < 50 bzw. < 40					
< 45 (yrs)	12.35	18.41	1.49 [1.15;1.93]	17.04	1.38 [0.98;1.95]
45–64 (yrs)	8.27	11.62	1.41 [1.04;1.89]	13.64	1.65 [1.21;2.24]
≥ 65(yrs)	8.13	10.46	1.29 [0.83;2.00]	12.36	1.52 [0.99;2.34]
Total	10.07	12.67	1.26 [1.06;1.50]	13.49	1.34 [1.12;1.61]
FPG > 100 mg/dl					
< 45 (yrs)	11.05	11.65	1.05 [0.69;1.60]	16.42	1.49 [0.92;2.39]
45–64 (yrs)	18.13	28.28	1.56 [1.23;1.99]	34.98	1.93 [1.52;2.45]
≥ 65(yrs)	32.92	37.35	1.13 [0.89;1.45]	46.37	1.41 [1.11;1.79]
Total	17.74	27.93	1.57 [1.34;1.85]	37.87	2.14 [1.83;2.50]
Smoker					
< 45 (yrs)	39.68	36.65	0.92 [0.80;1.06]	38.33	0.97 [0.80;1.16]
45–64 (yrs)	23.47	25.06	1.07 [0.90;1.26]	28.65	1.22 [1.03;1.45]
≥ 65(yrs)	7.17	10.24	1.43 [0.89;2.28]	10.07	1.41 [0.88;2.25]
Total	27.97	22.91	0.82 [0.74;0.91]	21.90	0.78 [0.70;0.88]
MI family history					
< 45 (yrs)	20.06	21.09	1.02 [0.82;1.27]	19.71	0.97 [0.71;1.31]
45–64 (yrs)	22.41	22.74	1.05 [0.88;1.25]	22.09	1.04 [0.86;1.27]
≥ 65(yrs)	14.45	12.16	0.86 [0.60;1.21]	15.66	1.10 [0.79;1.53]
Total	20.03	19.25	0.98 [0.87;1.11]	19.08	1.00 [0.87;1.15]
Diabetes					
< 45 (yrs)	1.57	3.05	1.94 [0.93;4.03]	3.91	2.49 [1.06;5.84]
45–64 (yrs)	10.39	15.12	1.45 [1.12;1.88]	21.61	2.08 [1.61;2.69]
≥ 65(yrs)	18.44	27.18	1.47 [1.13;1.93]	32.61	1.77 [1.36;2.30]
Total	7.78	16.39	2.11 [1.75;2.53]	24.24	3.11 [2.60;3.74]
Number of risks	Mean	Mean		Mean	
< 45 (yrs)	2.68	3.09		3.00	
45–64 (yrs)	3.37	3.51		3.39	
≥ 65(yrs)	3.90	4.03		3.83	
Total	2.88	3.44		3.93	

Cardiovascular risk factors are increased in male overweight patients with a high waist circumference (WC). PRR mean prevalence rate ratio; CI, confidence interval; FPG, fasting plasma glucose; MI, myocardial infarction. * the mean number of risks reflects the number of risk factors positive from the above mentioned.

**Table 3:** Cardiovascular risk factors in overweight women split into WC categories

	Women BMI ≥ 25 & < 30 kg/m^2^				
	≤ 80 cm	> 80 & ≤ 88 cm		> 88 cm	

	%	%	PRR [95%CI]	%	PRR [95%CI]

LDL > 115 mg/dl					
< 45 (yrs)	44.47	53.57	1.21 [1.06;1.37]	57.47	1.29 [1.14;1.47]
45–64 (yrs)	68.68	73.81	1.07 [0.99;1.16]	74.92	1.10 [1.01;1.19]
≥ 65(yrs)	71.23	72.38	1.02 [0.91;1.14]	72.27	1.01 [0.91;1.13]
Total	58.44	67.33	1.15 [1.08;1.23]	70.69	1.21 [1.14;12.8]
TG > 150 mg/dl					
< 45 (yrs)	13.37	21.40	1.60 [1.20;2.14]	32.20	2.41 [1.83;3.18]
45–64 (yrs)	21.26	31.14	1.46 [1.17;1.83]	42.48	2.00 [1.62;2.47]
≥ 65(yrs)	35.62	37.33	1.05 [0.82;1.34]	47.21	1.33 [1.06;1.66]
Total	20.16	29.68	1.47 [1.27;1.70]	42.39	2.10 [1.83;2.41]
RR ≥ 140/90 mmHg					
< 45 (yrs)	4.35	7.50	1.72 [1.01;2.95]	14.29	3.29 [1.99;5.42]
45–64 (yrs)	20.17	23.05	1.14 [0.90;1.45]	29.94	1.48 [1.19;1.85]
≥ 65(yrs)	32.65	37.69	1.15 [0.89;1.49]	41.37	1.27 [1.00;1.61]
Total	15.28	21.85	1.43 [1.20;1.70]	31.44	2.06 [1.75;2.42]
Low phys. activity					
< 45 (yrs)	73.25	73.83	1.01 [0.93;1.09]	78.73	1.07 [1.00;1.16]
45–64 (yrs)	68.70	74.09	1.08 [1.00;1.69]	77.84	1.13 [1.05;1.22]
≥ 65(yrs)	76.92	78.81	1.03 [0.93;1.13]	84.63	1.10 [1.00;1.21]
Total	72.05	75.12	1.04 [0.99;1.09]	80.55	1.12 [1.07;1.17]
HDL < 50 bzw. < 40					
< 45 (yrs)	10.54	15.93	1.51 [1.08;2.12]	23.61	2.24 [1.63;3.09]
45–64 (yrs)	7.47	11.73	1.57 [1.04;2.36]	15.66	2.10 [1.42;3.09]
≥ 65(yrs)	17.81	11.64	0.65 [0.43;1.00]	19.11	1.07 [0.74;1.55]
Total	10.53	12.98	1.23 [0.99;1.54]	18.45	1.75 [1.43;2.15]
FPG > 100 mg/dl					
< 45 (yrs)	3.85	3.30	0.86 [0.31;2.36]	5.48	1.43 [0.57;3.54]
45–64 (yrs)	9.68	15.61	1.61 [0.95;2.73]	21.79	2.25 [1.37;3.71]
≥ 65(yrs)	23.68	26.39	1.11 [0.71;1.74]	34.81	1.47 [0.97;2.23]
Total	10.08	15.36	1.52 [1.10;2.12]	24.32	2.41 [1.77;3.28]
Smoker					
< 45 (yrs)	30.13	32.52	1.08 [0.89;1.30]	37.42	1.24 [1.04;1.49]
45–64 (yrs)	17.40	19.90	1.14 [0.88;1.49]	24.97	1.43 [1.12;1.84]
≥ 65(yrs)	5.00	4.23	0.85 [0.37;1.95]	6.16	1.23 [0.58;2.61]
Total	21.06	20.14	0.96 [0.82;1.12]	20.22	0.96 [0.83;1.11]
MI family history					
< 45 (yrs)	26.98	25.00	0.93 [0.75;1.15]	26.67	0.97 [0.78;1.19]
45–64 (yrs)	29.28	28.37	0.98 [0.80;1.19]	28.96	0.99 [0.82;1.20]
≥ 65(yrs)	20.16	18.10	0.86 [0.59;1.27]	18.84	0.95 [0.67;1.36]
Total	26.75	24.94	0.93 [0.81;1.07]	24.80	0.94 [0.83;1.07]
Diabetes					
< 45 (yrs)	0.51	1.05	2.06 [0.43;9.88]	1.99	3.91 [0.89;17.23]
45–64 (yrs)	4.86	6.07	1.25 [0.74;2.11]	12.34	2.54 [1.57;4.11]
≥ 65(yrs)	13.61	18.03	1.33 [0.85;2.07]	26.65	1.96 [1.30;2.97]
Total	4.39	7.42	1.69 [1.20;2.38]	15.89	3.62 [2.64;4.97]
Number of risks	Mean	Mean		Mean	
< 45 (yrs)	2.25	2.51		3.00	
45–64 (yrs)	2.59	2.92		3.25	
≥ 65(yrs)	3.23	3.58		3.79	
Total	2.41	2.90		3.58	

Cardiovascular risk factors are increased in female overweight patients with a high waist circumference (WC). PRR mean prevalence rate ratio; CI, confidence interval; FPG, fasting plasma glucose; MI, myocardial infarction. * the mean number of risks reflects the number of risk factors positive from the above mentioned.

The relation of WC to high LDL-cholesterol, low physical activity and low HDL-cholesterol was not uniform throughout age groups and genders. An elevated LDL-cholesterol (> 115 mg/dl) was slightly more frequent in patients with a high WC, but not in all age groups. The same was true for low physical activity. A low HDL-cholesterol was more prevalent in patients with a high WC except in the elderly (≥ 65 years).

### PROCAM and SCORE Score per BMI and WC

In Table 4 and 5, BMI and WC categories are displayed against each other and are stratified by gender. Table 4 reports the PROCAM Score (risk of a cardiovascular event within the next 10 years). It shows that there was a steady increase in risk in both genders from the upper left hand (low BMI and WC) to the lower right hand (high BMI and high WC; mean adjusted risk in male 6.18%, mean adjusted risk in female 1.82%). Furthermore, the risk in the highest risk category in females (max. 1.82%) was even lower than in the lowest male risk category (min. 3.78%). The same was true for the SCORE Score (risk of cardiovascular death within the next 10 years). Again, patients with a high BMI and high WC had the highest risk (male adjusted max. 2.61%; female adjusted max. 1.63). Overall female patients were at a substantially lower risk (max. 1.63%) than male patients (min. 1.90%).

**Table 4:** PROCAM risk crude (and standardized for age differences in brackets) in relation to BMI and waist circumference.

A. Male	≤ 94 cm	> 94 & ≤ 102 cm	> 102 cm	Total
< 25 kg/m^2^	3.61 (3.78) [n = 1,800; 23.41%]	5.84 (4.91) [n = 296; 3.85%]	6.77 (4.63*) [n = 30; 0.39%]	3.97 (4.0) [n = 2,126; 27.65%]
≥ 25& < 30 kg/m^2^	4.56 (4.64) [n = 1,026; 13.34%]	6.49 (5.49) [n = 1,564; 20.34%]	7.68 (5.83) [n = 974; 12.67%]	6.26 (5.35) [n = 3,564; 46.35%]
≥ 30 kg/m^2^	6.15 (5.9*) [n = 28; 0.36%]	6.07 (5.96) [n = 230; 2.99%]	7.35 (6.18) [n = 1,742; 22.65%]	7.19 (6.16) [n = 2,000; 26.01%]
Total	3.98 (4.12) [n = 2,854; 37.11%]	6.35 (5.47) [n = 2,090; 27.18%)	7.46 (6.05) [n = 2,746; 35.71%)	5.87 (5.22) [7,690; 100.00%]

B. Female	≤ 80 cm	> 80 & ≤ 88 cm	> 88 cm	Total

< 25 kg/m^2^	0.94 (1.08) [n = 2,438; 27.50%]	1.35 (1.35) [n = 889; 10.03%]	1.63 (1.62) [n = 223; 2.52%]	1.09 (1.19) [n = 3,550; 40.05%]
≥ 25& < 30 kg/m^2^	1.15 (1.23) [n = 360; 4.06%]	1.43 (1.46) [n = 1,008; 11.37%]	1.85 (1.75) [n = 1,566; 17.66%]	1.62 (1.59) [n = 2,934; 33.10%]
≥ 30 kg/m^2^	1.37 (1.29*) [n = 13; 0.15%]	1.53 (1.5) [n = 96; 1.08%]	1.86 (1.82) [n = 2,272; 25.63%]	1.85 (1.81) [n = 2,381; 26.86%]
Total	0.97 (1.1) [n = 2,811; 31.71%]	1.40 (1.41) [n = 1,993; 22.48%]	1.85 (1.78) [n = 4,061; 45.81%]	1.47 (1.5) [8865; 100.00%]

[number of patients per patient group with non-missing PROCAM; proportion of all patients with non-missing PROCAM].

PROCAM risk in relation to BMI and waist circumference [12]. N means number of patients with non-missing PROCAM in each cell; % of inner 9 cells adds up to 100%; the figure displayed is the risk for cardiovascular events for the next 10 years calculated with the PROCAM score (standardized for age). Crude/age-standardized mean are reported. * means that the total N in this cell is 30 or below and therefore the direct age standardization may have led to unreliable results.

**Table 5:** SCORE Score in relation to BMI and waist circumference.

A. Male	≤ 94 cm	> 94 & ≤ 102 cm	> 102 cm	Total
< 25 kg/m^2^	1.86 (1.90) [n = 1,801; 23.40%]	3.01 (2.25) [n = 295; 3.83%]	3.77 (2.19) [n = 31; 0.40%]	2.05 (1.97) [n = 2,127; 27.63%]
≥ 25 & < 30 kg/m^2^	1.99 (2.03) [n = 1024; 13.30%]	2.98 (2.32) [n = 1,578; 20.50%]	3.62 (2.45) [n = 977; 12.69%]	2.87 (2.29) [n = 3,579; 46.49%]
≥ 30 kg/m^2^	2.22 (2.31*) [n = 27; 0.35%]	2.67 (2.57) [n = 231; 3.00%]	3.42 (2.61) [n = 1,734; 22.53%]	3.32 (2.6) [n = 1,992; 25.88%]
Total	1.91 (1.95) [n = 2,852; 37.05%]	2.95 (2.33) [n = 2,104; 27.33%]	3.50 (2.55) [n = 2,742; 35.62%]	2.76 (2.29) [7,698; 100.00%]

B. Female	≤ 80 cm	> 80 & ≤ 88 cm	> 88 cm	Total

< 25 kg/m^2^	0.95 (1.18) [n = 2,585; 28.15%]	1.32 (1.30) [n = 948; 10.32%]	1.61 (1.52) [n = 235; 2.56%]	1.09 (1.24) [n = 3,768; 41.03%]
≥ 25 & < 30 kg/m^2^	1.07 (1.16) [n = 365; 3.97%]	1.38 (1.37) [n = 1031; 11.23%]	1.82 (1.63) [n = 1,600; 17.42%]	1.58 (1.5) [n = 2,996; 32.63%]
≥ 30 kg/m^2^	1.50 (1.71*) [n = 14; 0.15%]	1.30 (1.27) [n = 98; 1.07%]	1.73 (1.63) [n = 2,307; 25.12%]	1.71 (1.62) [n = 2,419; 26.34%]
Total	0.97 (1.18) [n = 2,964; 32.28%]	1.35 (1.34) [n = 2,077; 22.62%]	1.76 (1.63) [n = 4,142: 45.11%]	1.41 (1.43) [9,183; 100.00%]

[number of patients per patient group with non-missing SCORE; proportion of all patients with non-missing SCORE].

SCORE Score in relation to BMI and waist circumference [13]. N means number of patients in each cell with non-missing SCORE; % of inner 9 cells adds up to 100%; the figure displayed is the risk for cardiovascular death for the next 10 years calculated with the SCORE score. Crude/age-standardized mean are reported. * means that the total N in this cell is 30 or below and therefore the direct age standardization may have led to unreliable results.).

## Discussion

Epidemiological research in primary care is, different from population-based samples, highly relevant when investigating topics related to physician – patient interaction. Primary care in Germany is characterized by a high patient load of about 73 consultations per day [16], which is much higher than in other countries in Europe and worldwide. Straight and easy to follow rules for screening and detection of cardiovascular disease are warranted to ensure, that busy clinical routine does not detract from cardiovascular prevention and chronic disease treatment. Obesity and in particular abdominal obesity has been recognized to be linked to an increase in cardiovascular risk and therefore the present analysis on the interrelationship of both measures and their relation to cardiovascular risk has been conducted.

The present analysis, based on data derived from more than 35,869 adult primary care attendees, uncovered several key aspects of overweight and obesity in primary care: 1) it documents a very high prevalence of overweight (36.5%) and obesity (23.9%) and of an increased and high WC in primary care practice in 2005, 2) it shows that a high waist circumference identifies patients with an increased cardiovascular risk even within the normal weight, overweight and obese patient group, 3) it confirms a tight relationship between waist circumference and an increase in cardiovascular risk and 4) it highlights a remarkable risk increase with BMI, WC and the combination of both measures.

### Prevalence of (abdominal) obesity in adults seeking primary care

Although difficult to compare with other studies due to considerable a difference in methods and sampling the present data indicate a high prevalence of overweight and obesity in primary health care in Germany. According to the "Shape of the Nations survey" 39% of all people visiting a primary care physicians worldwide were overweight or obese; in North America, this proportion was 49% [17]. In the Netherlands, a population closely resembling the German population, obesity was observed in 8.9% of men and 12.4% of women; for overweight these percentages were 42.2% and 30.4%, respectively [18]. On the other hand, in primary care of the southeastern United States (1999–2002) substantially higher prevalence rates for overweight (white women 26%, men 24%) and obesity (white women 36%, men 32%) have been reported [19]. Previous data from German primary care are available for 2001 from the HYDRA- and for 2003 from the DETECT-study [4,20]. Prevalence rates in 2001 were 37.9% for overweight and 19.4% for obesity, prevalence rates in 2003 were 37.3% for overweight and 22.5% for obesity.

36.4% of male and 41.5% of female patients in our primary care cohort had a high WC (> 102 cm men, > 88 cm women). This appears high as compared to population-based samples across Europe [21-26], which one would expect based on the higher morbidity in a primary care sample. It is lower, however, as compared to the recent nationwide DETECT study of 55,518 consecutive German primary care attendees. In this survey, 43% of male and 53% of female patients met criteria for abdominal obesity (waist circumference ≥ 102 for men and ≥ 88 for women) [20]. Methodological differences between the GEMCAS and the DETECT cohort may account for differences in prevalence rates reported. For example, GEMCAS excluded physicians specialized in diabetology or cardiology in an attempt to document prevalence rates in a broad sample of primary care attendees not being confounded by a large group of multimorbid patients attending a specialized physician. Secondary to this, the difference may be related to the fact that more diabetic patients were included in DETECT (14.6% vs. 12.4%) and patients were slightly older 53.9 vs. 51.7 years. Data from another source became recently available from the global IDEA study in primary care in 63 countries [27,28]. The data show a fairly consistent prevalence of overweight all over the world with substantial differences in obesity prevalence. Whether this observation could also be due to different BMI thresholds that may apply in particularly Asian countries is a matter of debate.

### Waist circumference in the overweight

There is some confusion both from the physician and patient perspective as to being overweight measured by BMI indicates an increased cardiovascular risk. A modest elevation of BMI may be simply due to a higher muscle mass, at least in younger male patients. We were therefore interested to assess if and to what extent WC is suitable to discriminate between normal and elevated risk. The data from the present study clearly show that even in the subgroup of overweight as well as in the subgroup of normal weight patients there is a proportion of patients with an elevation of waist circumference and an unfavourable cardiovascular risk profile. This could guide both physicians and patients towards more aggressively lowering increased body fat in high risk patients with overweight also.

Earlier studies have focussed on whether BMI, abdominal obesity or measures like waist-to-height ratio (WHtR) or waist-to-hip ratio (WHR) may be best to describe an excess of fat and its relation to cardiovascular risk [1]. Several studies have described that WC is a better predictor of cardiovascular risk outcome than BMI [5-8]. While this debate is very valid, the mostly used measure of obesity in primary care is still BMI for which both components (weight, height) are easy and not offensive to determine. Given this standard, it is reassuring to know that normal-weight patients generally have a normal WC and a normal cardiovascular risk. On the other hand, obese persons have a high WC and a high cardiovascular risk. For the group of patients in between, however, it is necessary to measure WC to determine body fat distribution, since 31% of male and 54% of female patients have a high WC when in the overweight category by BMI. As shown in Table 2 &3 most risk factors are significantly elevated in overweight patients with a high WC, the high cut point showing the highest PRRs (stratified into age group and gender). Tables 4 and 5 nicely illustrate that it is particularly hazardous to have a low to moderate BMI but a high degree of abdominal obesity. A higher BMI with a low waist circumference on the other hand may point at individuals with either a more favourable fat distribution or a higher muscle mass (particularly in overweight male patients).

### Strength and limitations

Despite the strengths of the study (sample size, representativeness for patients in primary care, simultaneous coverage of structural, doctors and patients perspective) two limitations need to be highlighted: While the data set is representative for the primary care population it may be less so for the general population. The degree of representativeness of the GEMCAS sample for primary care has been reported in more detail by Moebus et al. [11]. In short, 2,600 out of the 17,271 initially contacted primary health care practices responded to the invitation to participate in the study (15% response). The response rate varied slightly in the different postal code areas but no differences greater than ± 2% between contacted and participating sites for each region could be observed. The first 2,070 response faxes were collected and analyzed for eligibility. These revealed 1,835 eligible physicians out of which the first 1,700 were recruited for participation. Among these, 140 cancelled their participation before starting the study, mainly due to time-related issues, communication problems or illness. 33 of the 1,835 originally eligible physicians were recruited in a second run to replace cancellations among the 1,700. The second limitation was that the Body Mass Index was self-reported in 2/3^rd ^of the patients in GEMCAS. This may indicate, that the prevalences reported are likely lower bound estimates for the true prevalence in this population [29].

## Conclusion

There is a high prevalence of obesity and in particular abdominal obesity among patients in primary health care in Germany. The determination of the BMI is sufficient to assess cardiovascular risk in obese patients (because BMI and WC measurements generally match well in these patients groups). Using WC however allows to identify high risk patients from within the overweight and even normal weight patient group. This approach may help to build on the educational activities to establish BMI as a marker for obesity and allows the more appropriate counselling in patients whose overweight is actually abdominal obesity.

## Competing interests

The authors declare that they have no competing interests.

## Authors' contributions

SM planned and performed the study. CL participated in the design of survey instruments and performed the statistical analysis. HH and PB have been writing the manuscript, HS and ES–T revised the manuscript for important intellectual content, JW participated in the study design. K–HJ supervised scientific, ethical and data privacy issues of the study. All authors read and approved the final manuscript.

## Pre-publication history

The pre-publication history for this paper can be accessed here:


